# Aluminous hydrous magnesium silicate as a lower-mantle hydrogen reservoir: a role as an agent for material transport

**DOI:** 10.1038/s41598-022-07007-8

**Published:** 2022-03-04

**Authors:** Akihiko Nakatsuka, Akira Yoshiasa, Makio Ohkawa, Eiji Ito

**Affiliations:** 1 Graduate School of Sciences and Technology for Innovation, Yamaguchi University, Ube, 755-8611 Japan; 2 Faculty of Advanced Science and Technology, Kumamoto University, Kumamoto, 860-8555 Japan; 3 Graduate School of Advanced Science and Engineering, Hiroshima University, Higashi-Hiroshima, 739-8526 Japan; 4 Institute for Planetary Materials, Okayama University, Misasa, 682-0193 Japan

**Keywords:** Mineralogy, Geochemistry, Geodynamics, Solid Earth sciences, Geophysics, Solid-state chemistry

## Abstract

The potential for storage of a large quantity of water/hydrogen in the lower mantle has important implications for the dynamics and evolution of the Earth. A dense hydrous magnesium silicate called phase D is a potential candidate for such a hydrogen reservoir. Its MgO–SiO_2_–H_2_O form has been believed to be stable at lower-mantle pressures but only in low-temperature regimes such as subducting slabs because of decomposition below mantle geotherm. Meanwhile, the presence of Al was reported to be a key to enhancing the thermal stability of phase D; however, the detailed Al-incorporation effect on its stability remains unclear. Here we report on Al-bearing phase D (Al-phase D) synthesized from a bridgmanite composition, with Al content expected in bridgmanite formed from a representative mantle composition, under over-saturation of water. We find that the incorporation of Al, despite smaller amounts, into phase D increases its hydrogen content and moreover extends its stability field not only to higher temperatures but also presumably to higher pressures. This leads to that Al-phase D can be one of the most potential reservoirs for a large quantity of hydrogen in the lower mantle. Further, Al-phase D formed by reaction between bridgmanite and water could play an important role in material transport in the lower mantle.

## Introduction

Water/hydrogen is transported into the Earth’s interior via hydrous mineral phases in subducting slabs, which affects melting^1^ in the Earth and its rheology^2–4^. Hydrous phases as potential water/hydrogen reservoirs especially in the lower mantle have important implications for the dynamics and evolution of the Earth. A number of high-pressure studies^5–16^ have demonstrated that several dense hydrous magnesium silicates (DHMS) are such potential candidates. Among DHMS phases, phase D (simplified formula MgSi_2_O_6_H_2_), identical to the later reported phase F and phase G, had been considered the highest-pressure phase. Later, the first principles simulations^11^ and high-pressure experiments^12^ reported the presence of phase H (simplified formula MgSiO_4_H_2_), a new DHMS stable at pressures higher than the stability field of phase D. A series of high-pressure experiments^13^ has demonstrated, however, that the MgO–SiO_2_–H_2_O forms of these DHMS phases are stable at slab temperatures but decompose at lower temperatures than the normal mantle-geotherm.

On the other hand, the high-pressure experiments^8^ conducted in a bulk composition adding 1 mass% Al_2_O_3_ component to the simplified phase D composition reported that phase D crystallized with several mol% Al_2_O_3_ component and broke down at ~ 1600 °C, higher temperature than in Al-free phase D, at 24 GPa. Super-aluminous phase D with extremely high Al-content (Mg_0.2_Fe_0.15_Al_1.8_SiO_6_H_1.8_)^10^ was first synthesized at 1300 °C and 25 GPa from a bulk composition (including 9.8 mass% H_2_O component) similar to that reported for bridgmanite formed from a mid-ocean ridge basalt (MORB) composition. The further experiment^10^ using the composition of this super-aluminous phase D produced the sample with the higher H-content (Mg_0.2_Fe_0.12_Al_1.5_Si_0.92_O_6_H_3.1_). Later, the high-pressure experiments^9^ in the simplified system Al_2_O_3_–SiO_2_–H_2_O reported that (Mg, Fe)-free super-aluminous phase D (simplified formula Al_2_SiO_6_H_2_) could be stable over 2000 °C at 26 GPa. Phase H can also incorporate a large amount of Al, forming a solid solution with δ-AlOOH^15,16^. The high-pressure experiments^16^ conducted in a bulk composition of 0.70MgSiO_3_·0.30Al_2_O_3_ with 1.5–7.0 mass% H_2_O component showed that the aluminous phase H (Al-phase H) was produced with more than 50 mol% Al_2_O_4_H_2_ component and could be stable even along the normal lower-mantle geotherm at > 40 GPa. In addition, these experiments showed that it could be stable to ~ 130 GPa, corresponding to a pressure at the lowermost mantle, along a subducting slab geotherm. Thus, the presence of Al is a potential key-factor for enhancing the thermal stability of these DHMS phases at the lower-mantle pressures. The recent high-pressure experiments^16–18^ demonstrated that Al ions are much more preferentially partitioned into hydrous phases (phase D or phase H) than anhydrous phases (bridgmanite or post-perovskite phase); this situation was observed even in Al-poor bulk-compositions^17^ such as peridotitic (or pyrolitic) composition. This suggests that under the presence of suitable water amount, the aluminous hydrous phases with high stability could exist not only in Al-rich fields such as MORB of subducting slabs but also everywhere in the lower mantle. However, the crystal-chemical mechanism for the stability enhancement of these hydrous phases due to the incorporation of Al remains to be solved. Here we report on Al-bearing phase D (Al-phase D) synthesized from high-pressure experiments of a bridgmanite composition in the system MgO–SiO_2_–Al_2_O_3_, with Al content close to that reported for bridgmanite formed from a representative mantle composition, under over-saturation of water. We reveal the incorporation mechanism of Al and a large amount of hydrogen (H) into phase D and demonstrate the drastic enhancement in stability of phase D due to the incorporation of a relatively small amount of Al. We discuss the mechanism for such a high stability of Al-phase D, in terms of crystal chemistry based on single-crystal X-ray diffraction. On the basis of these findings, we propose crucial implications for the recycle of water in the lower mantle.

We selected the starting composition of 0.92MgSiO_3_·0.08Al_2_O_3_ because it is close to the composition of bridgmanite, 0.94MgSiO_3_·0.06Al_2_O_3_ (Refs.^19,20^), expected in a pyrolitic^21^ lower-mantle. Our high-pressure experiments were conducted under the three different thermal histories. Their experimental conditions are summarized in Table 1. Reagent grade oxides and hydroxides were mixed in the required ratios and sealed in platinum (Pt) capsules together with amounts of liquid water suitable for over-saturation. The samples were compressed to 27 GPa (runs #1 and #2) or 26 GPa (run #3) and then heated to each target maximum temperature of 1600 °C (run #1) or 1900 °C (runs #2 and #3) using a Kawai-type multi-anvil apparatus^22^. After undergoing each thermal history, the samples were quenched at 1600 °C (runs #1 and #2) or 1300 °C (run #3) and recovered to ambient conditions. In all the runs, liquid water was seeping out of the Pt capsules when those were opened, which shows that the recovered samples were synthesized under over-saturation of water.

**Table 1:** Conditions of the high-pressure experiments. *T*_*max*_ target maximum temperature, *T*_*q*_ quenching temperature.

Run no.	Starting materials	*P* (GPa)	*T*_max_ (°C)	Duration at *T*_max_ (min)	*T*_q_ (°C)	Cooling rate from *T*_max_ to *T*_q_ (°C/min)
#1	MgO, SiO_2_, Al(OH)_3_, H_2_O	27	1600	10	1600	No cooling
#2	Mg(OH)_2_, SiO_2_, Al(OH)_3_, H_2_O	27	1900	5	1600	50
#3	MgO, SiO_2_, Al(OH)_3_, H_2_O	26	1900	10	1300	30

**Figure 1:**
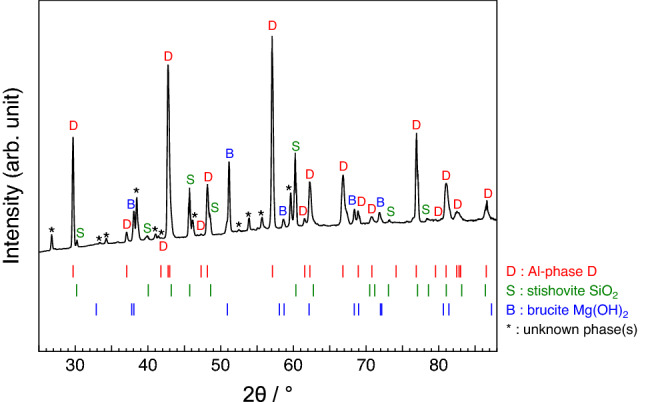
Microfocus powder X-ray diffraction pattern of a recovered sample. As an example, that of the run #2 is given here.

We measured the microfocus X-ray diffraction patterns for the recovered samples of the runs #1 and #2. A typical example of them is shown in Fig. 1. In the patterns, we observed the diffraction peaks corresponding to phase D, stishovite SiO_2_ and brucite Mg(OH)_2_. The peaks that cannot be assigned to any known-phases were also observed; these are probably due to impurities precipitated, together with brucite, from fluid during quenching. Here, we determined the unit-cell parameters with trigonal symmetry by least-squares fits of the *d* spacings of 21 peaks assigned to phase D as follows: *a* = 4.8239(1) Å, *c* = 4.3134(2) Å, *V* = 86.924(4) Å^3^ for the run #1; *a* = 4.8416(1) Å, *c* = 4.3236(2) Å, *V* = 87.771(4) Å^3^ for the run #2. The calculated *d* values of these peaks are in good agreement with the observed ones (Table 2).

**Table 2:** Microfocus X-ray diffraction data for Al-phase D in the recovered samples of the runs #1 and #2. Calculated unit-cell parameters and volumes: *a* = 4.8239(1) Å, *c* = 4.3134(2) Å, *V* = 86.924(4) Å^3^ for the run #1; *a* = 4.8416(1) Å, *c* = 4.3236(2) Å, *V* = 87.771(4) Å^3^ for the run #2.

Sample	Run #1				Run #2			
*hkl*	*I*/*I*_0_	*d*_obs_ (Å)	*d*_cal_ (Å)	*d*_obs_–*d*_cal_ (Å)	*I*/*I*_0_	*d*_obs_ (Å)	*d*_cal_ (Å)	*d*_obs_–*d*_cal_ (Å)
101	25	2.9964	3.0009	− 0.0045	47	3.0088	3.0100	− 0.0012
110	< 1	2.4169	2.4119	0.0050	5	2.4254	2.4208	0.0046
002	2	2.1543	2.1567	− 0.0024	< 1	2.1625	2.1618	0.0007
111	35	2.1096	2.1052	0.0044	83	2.1145	2.1123	0.0022
200	33	2.0932	2.0888	0.0044	38	2.1030	2.0965	0.0065
102	< 1	1.9177	1.9164	0.0013	< 1	1.9219	1.9214	0.0005
201	45	1.8803	1.8800	0.0003	26	1.8888	1.8864	0.0024
112	100	1.6074	1.6077	− 0.0003	100	1.6129	1.6124	0.0005
202	3	1.5036	1.5004	0.0032	4	1.5065	1.5050	0.0015
211	44	1.4892	1.4828	0.0064	23	1.4910	1.4880	0.0030
300	9	1.3914	1.3925	− 0.0011	35	1.4002	1.3977	0.0025
103	11	1.3588	1.3595	− 0.0007	8	1.3624	1.3629	− 0.0005
301	5	1.3298	1.3252	0.0046	5	1.3309	1.3299	0.0010
212	1	1.2732	1.2740	− 0.0008	< 1	1.2790	1.2781	0.0009
113	10	1.2351	1.2350	0.0001	28	1.2396	1.2384	0.0012
220	1	1.2084	1.2060	0.0024	< 1	1.2049	1.2104	− 0.0055
203	3	1.1858	1.1843	0.0015	24	1.1872	1.1876	− 0.0004
302	4	1.1653	1.1699	− 0.0046	9	1.1695	1.1737	− 0.0042
221	< 1	1.1595	1.1614	− 0.0019	< 1	1.1658	1.1656	0.0002
310	3	1.1549	1.1587	− 0.0038	3	1.1636	1.1629	0.0007
311	5	1.1199	1.1190	0.0009	12	1.1247	1.1230	0.0017

To confirm the presence of phase D, we conducted the electron probe microanalyses for the recovered sample of the run #1. The analytical result showed the presence of products with a chemical composition of 23.11 mass% MgO, 42.97 mass% SiO_2_ and 18.66 mass% Al_2_O_3_ with a total of 84.74 mass%. This phase was damaged by the electron beam. The deficiency (15.26 mass%) from 100 mass% is attributed to the incorporation of H_2_O component into the structure, and the chemical formula of the products was calculated to be Mg_1.01_Si_1.26_Al_0.65_O_6_H_2.99_. From the compatibility with phase D^5,23–25^ in terms of unit-cell parameters and chemical formula, it is concluded thus that the present hydrous phase is Al-phase D. These results obtained from the samples quenched at 1600 °C at 27 GPa, lying in the normal mantle-geotherm, substantiates that phase D is stable even at the conditions corresponding to the uppermost parts in the lower mantle if it contains some amount of Al_2_O_3_ component, in contrast to Al-free phase D, which decomposes at 1200 °C (Ref.^7^).

**Figure 2:**
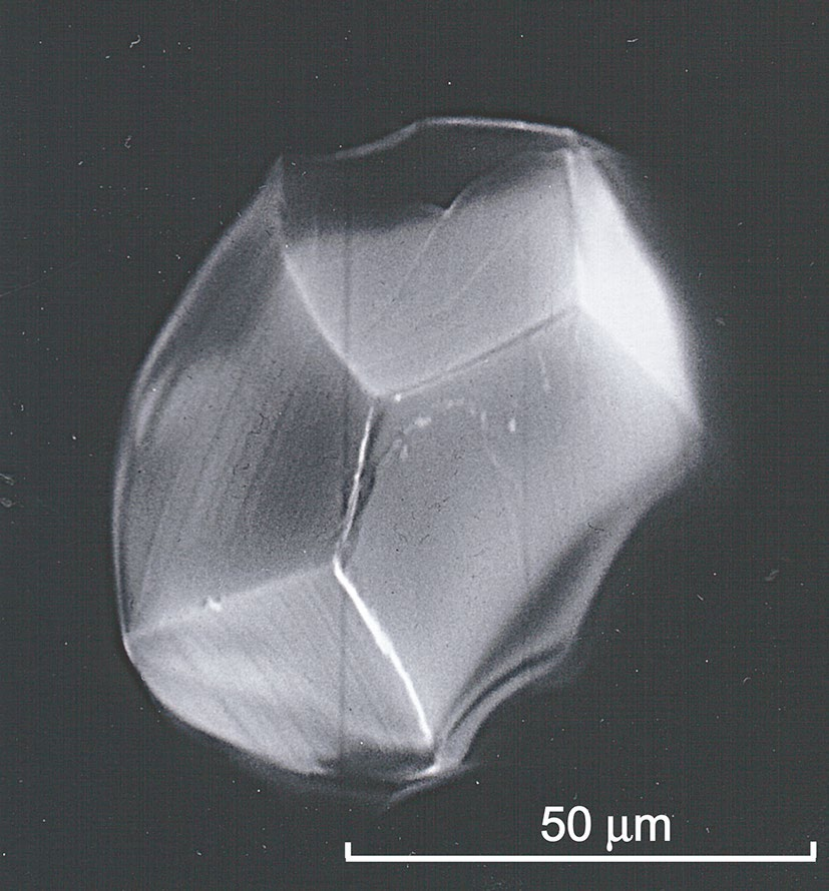
Scanning electron microscope image of a synthesized Al-phase D single-crystal. As an example, that of the run #3 is given here.

To assess why the presence of Al drastically enhances the stability of phase D, it is quite important to determine the detailed crystal structure of Al-phase D. The slow cooling from higher temperatures is effective to enhance crystal growth from melt, as in the case of Al-free MgSiO_3_ bridgmanite^26^. The runs #2 and #3, with the slow cooling from 1900 °C, were thus conducted to try synthesis of Al-phase D single-crystals large enough for single-crystal X-ray diffraction. Numerous transparent and euhedral single-crystals (Fig. 2), which possess a crystal habit implying a trigonal or a hexagonal symmetry, were found in the recovered samples. No intergrowth textures were observed under polarized microscope. A specimen for single-crystal X-ray diffraction was selected from the crystals produced in the run #3, with the slower cooling rate than in the run #2, because they were better in terms of size and crystallinity than those produced in the run #2. The electron probe microanalyses for the crystals produced in the run #3 showed a chemical composition of 28.46 mass% MgO, 48.69 mass% SiO_2_ and 7.72 mass% Al_2_O_3_ with a total of 84.87 mass% to give a chemical formula of Mg_1.25_Si_1.43_Al_0.27_O_6_H_2.97_ by assigning the deficit from 100 mass% to H_2_O component. This composition differs somewhat from that in the run #1 shown above. This is probably due to the difference in thermal history and/or water fugacity, which can influence Mg/Si ratio in fluid, between the two runs.

**Figure 3:**
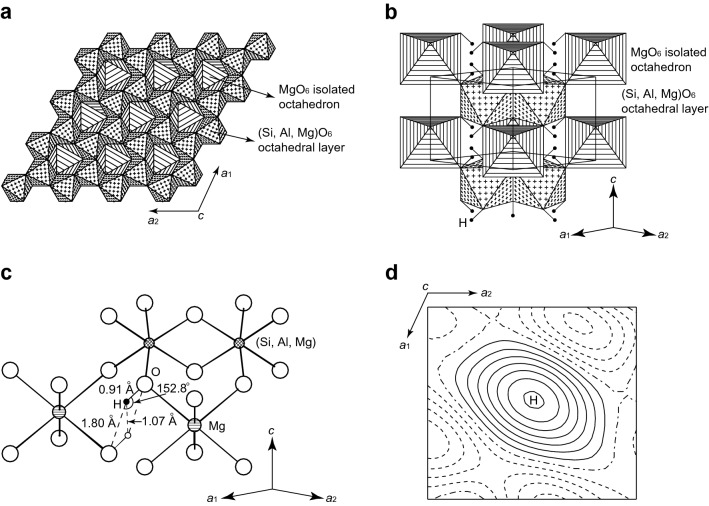
Crystal structure of the present Al-phase D, analyzed using a single-crystal produced in the run #3. (**a**) Projection along the *c*-axis (excluding H atoms). (**b**) Projection along the direction close to [110]. H site is denoted with small solid circles. In the present Al-phase D, H atoms occupy about 50% of this site. The crystal structure is based on the hexagonal closest packing array of O atoms and consists of the two types of octahedra, (Si, Al, Mg)O_6_-octahedra in S-site and MgO_6_-octahedra in M-site. The (Si, Al, Mg)O_6_-octahedra form the layer structure by sharing edges with each other, and the layers are stacked along the *c*-axis. The MgO_6_-octahedra, isolated with each other, connect the separations between the (Si, Al, Mg)O_6_-octahedral layers by sharing corners with the (Si, Al, Mg)O_6_-octahedra. The hydrogen bonds contribute to the linkages between the MgO_6_-octahedra and between the MgO_6_- and the (Si, Al, Mg)O_6_-octahedra. (**c**) Crystallographic configuration of H atom. The small solid circle is an occupied H-position, and the small open circle is an unoccupied H-position. (**d**) Difference Fourier map denoting the residual electron density peak assigned to a H atom. The contour interval is 0.05 eÅ^−3^. Positive contours are solid lines. Negative and zero contours are dashed lines and dashed-and-dotted lines, respectively.

The crystal structure determined for the selected crystal (run #3) are shown in Fig. 3a–c, together with the residual electron density peak (Fig. 3d) assigned to a hydrogen (H) atom. The structure-analytical information and results are given in Supplementary Table S1, and Tables 3 and 4. The M–O bond length [2.113(2) Å] in the present Al-phase D single-crystal (Mg_1.25_Si_1.43_Al_0.27_O_6_H_2.97_) agrees excellently with that [2.114(3) Å^3^] in the reported Al-free phase D (Mg_1.24_Si_1.76_O_6_H_2.48_)^24^, whereas the S–O bond length [1.840(1) Å] in the former is significantly larger than that [1.823(2) Å] in the latter. This shows that larger Al^3+^ is incorporated only into S-site and substitutes smaller Si^4+^, justifying the present site-assignment of Al. Indeed, bond valence sums^27^, calculated including H–O (donor) and H···O (acceptor) bonds, are 1.88 for M-site, 3.42 for S-site and 2.02 for O-site, approximately equal to their expected values (1.96, 3.54 and 2.0, respectively), demonstrating that the resulting positional parameters and site occupancy parameters are crystal-chemically reasonable.

**Table 3:** Refined structural parameters. *W.p.* Wyckoff position. ^a^Site occupancy parameters of Si and Al were fixed on S-site, and those of Mg were constrained between M- and S-sites to keep the chemical composition from the electron probe microanalyses. ^b^H-atom position was determined from the difference Fourier synthesis, and not refined.

Site (W.p.)	M (1*a*)	S (2*d*)	O (6*k*)	H (6*k*)^b^
Occupancy	0.979(8) Mg^a^	0.715 Si^a^	1.0	0.495
		0.135 Al^a^		
		0.135 Mg^a^		
*x*	0	0.3333	0.6340(5)	0.495
*y*	0	0.6667	0	0
*z*	0	0.5	0.2669(4)	0.124
*U*_eq_ (Å^2^)	0.0130(12)	0.0101(7)	0.0132(17)	–
*U*_11_ (Å^2^)	0.0136(12)	0.0108(7)	0.0128(12)	–
*U*_22_ (Å^2^)	0.0136	0.0108	0.0208(15)	–
*U*_33_ (Å^2^)	0.0118(16)	0.0085(9)	0.0085(11)	–
*U*_12_ (Å^2^)	0.0068	0.0054	0.0104	–
*U*_13_ (Å^2^)	0	0	− 0.0002(7)	–
*U*_23_ (Å^2^)	0	0	0	–

**Table 4:** Selected interatomic distances and angles.

Bonds/separations	Distances (Å)	Angles (°)
M–O	2.113(2) × 6	–
S–O	1.840(1) × 6	–
H–O (donor)	0.913	–
H···O (acceptor)	1.802	–
H···H	1.073	–
O–H···O	–	152.84

Owing to the constraints of the space group, a pair of centrosymmetric H positions (Wyckoff position 6*k*) are present in close proximity (H···H = 1.07 Å) (Fig. 3c, Table 4). If an H position is occupied, its nearest H position must be unoccupied to avoid an H^+^–H^+^ interaction (Fig. 3c). Thus, the maximum allowance for the number of H atoms contained in a unit cell is 3, corresponding to a half occupancy of the 6*k*-site. The H content of the present Al-phase D (2.97–2.99 H atoms per unit cell) is very close to the maximum allowance. The comparison of the cation ratios of Al-free phase D (Mg_1.11–1.24_Si_1.73–1.89_O_6_H_2.22–2.81_)^5,23–25^ with those of the present Al-phase D shows that the substitution Si^4+^ → Al^3+^ + H^+^, in addition to Si^4+^ → Mg^2+^ + 2H^+^ and Mg^2+^ → 2H^+^  + Vc (Vc: cation vacancy) suggested for Al-free phase D^5^, is responsible for a larger amount of H in Al-phase D. The contents of Al^3+^, Si^4+^, Mg^2+^ and Vc in Al-phase D are adjusted under the constraint that the number of H^+^ per unit cell must be equal to or less than 3.

**Figure 4:**
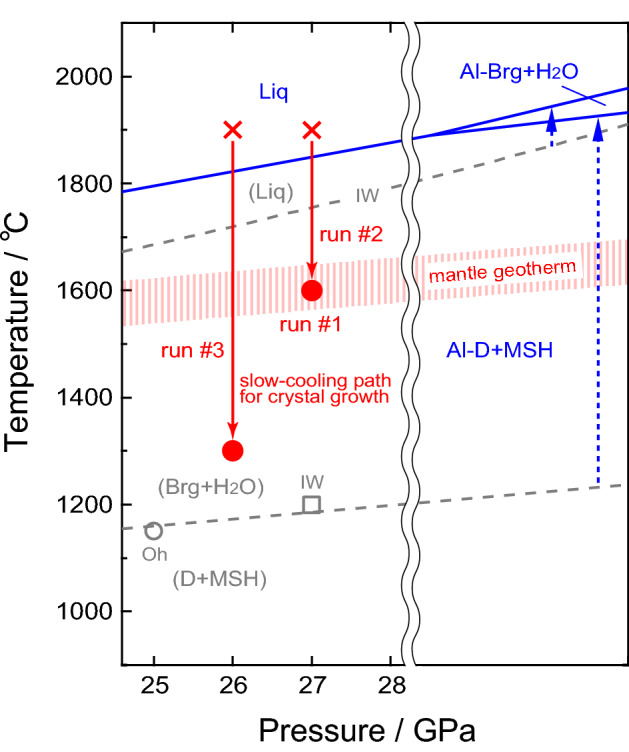
Possible stability relations under water-saturated conditions for the MgSiO_3_ system (dotted grey lines) and for the Al_2_O_3_-bearing MgSiO_3_ system (solid blue lines) inferred from the present results and the previous studies^6,26^. For the Al_2_O_3_-bearing MgSiO_3_ system, about 8 mol% of Al_2_O_3_ component is assumed implicitly. Phase abbreviations: D, phase D; Brg, MgSiO_3_ bridgmanite; Al-D, Al-bearing phase D; Al-Brg, Al-bearing MgSiO_3_ bridgmanite; Liq, liquid; MSH, residual MgO–SiO_2_–H_2_O components. Stability fields with and without parentheses denote those for the Al_2_O_3_-free and the Al_2_O_3_-bearing systems, respectively. The open grey circle, Oh, and the open grey square, IW, show stable existences of D + MSH (Ref.^6^) and Brg + H_2_O (Ref.^26^) in the Al_2_O_3_-free system, respectively, which could constrain the phase boundary between D + MSH and Brg + H_2_O (in this case, MSH was identified as superhydrous phase B, Mg_10_Si_3_O_18_H_4_, by Ref.^6^). The liquidus of MgSiO_3_ is quoted from Ref.^26^. The red crosses and the solid red arrows represent the soaking temperature (1900 °C) and the slow-cooling paths (from 1900 to 1600 °C at 27 GPa and from 1900 to 1300 °C at 26 GPa) in our crystal-growth experiments, respectively. The long and short dotted blue arrows show possible shifts of the boundary between D + MSH and Brg + H_2_O and the liquidus toward high temperature by incorporating Al_2_O_3_ component, respectively. The hatched pale-red zone represents the normal mantle-geotherm. Remark: Both melting and dehydration of dense substances are endothermic (Δ*H* > 0, i.e., Δ*S* = Δ*H*/*T* > 0) and generally accompany the increase in total volume (Δ*V* > 0) owing to the large volumes of the melts or the released water. Thus, positive Clapeyron slopes (d*T*/d*P* = Δ*V*/Δ*S* > 0) are adopted for all the phase boundaries.

In Fig. 4, possible phase relations for pure MgSiO_3_ (dotted grey lines) and Al_2_O_3_-bearing MgSiO_3_ (solid blue lines) under water-saturated conditions are shown together with the slow-cooling paths (solid red arrows) adopted for our crystal-growth experiments. As the liquidus temperature of MgSiO_3_ at 27 GPa under water saturation is 1750 °C (Ref.^26^), our charges would have been above liquidus when kept at 1900 °C in the crystal-growth experiments, in spite of a slight increase of the liquidus due to the Al_2_O_3_ component^28^. Thus, it is inferred that the single crystals of Al-phase D grew from melt. This view is supported by the fact that the crystals have a perfect euhedral-shape (see Fig. 2) and exhibit no intergrowth texture; these observations exclude the possibility that the crystals are product of reaction involving any other phases, such as Al-bearing MgSiO_3_ bridgmanite (Al-Brg). It is therefore concluded that Al-phase D is stable up to temperatures substantially higher than the normal mantle-geotherm. Moreover, as Al-phase D crystallized from a starting material with the composition of Al-Brg, the former should be more stable than the latter under water-saturated conditions.

Such a drastic change in stability relations between bridgmanite and phase D under water-saturated conditions by addition of a relatively small amount of Al_2_O_3_ component is interpreted in terms of the difference in the coordination environments of Al ions between Al-Brg and Al-phase D. The Al ions in Al-Brg occupy both the eightfold- and sixfold-coordinated sites by the substitution ^VIII^Mg^2+^ + ^VI^Si^4+^ → ^VIII^Al^3+^ + ^VI^Al^3+^ (Ref.^29^). Eightfold coordination is unsuitable for Al^3+^ because of its small cationic size; indeed, no compound with eightfold-coordinated Al is known except for Al-Brg. Such an unusual eightfold-coordinated Al should enhance the cohesive energy of Al-Brg, and the repulsive interaction with adjacent Si^4+^ or Al^3+^ through the shared faces between (Si, Al)O_6_-octahedra and (Mg, Al)O_8_-polyhedra may especially tend to destabilize the structure. In contrast, the incorporation of Al into phase D will reduce the cation-cation repulsion across the shared edges between (Si, Al, Mg)O_6_-octahedra in S-site (Fig. 3a,b) by the substitution of Al^3+^ for Si^4+^, owing to the lower charge and larger cationic size of Al^3+^. Thus, the incorporation of Al into phase D results in expansion of the stability field to much higher temperatures and presumably to much higher pressures.

It follows from this discussion that phase D could be stable along the normal lower-mantle geotherm up to much higher pressures if it contains some amount of Al_2_O_3_ component (cf. Fig. 4). Bridgmanite in the lower mantle probably contains about 6 mol% Al_2_O_3_ component^19,20^, and Al-phase D in the lower mantle could form by reaction between bridgmanite and free water. Given that the representative formula of Al-phase D is MgSi_2–*x*_Al_*x*_O_6_H_2+*x*_, the reaction can be expressed as follows:

1 $$\begin{aligned} &x{\text{Mg}}_{{0.94}} {\text{Si}}_{{0.94}} {\text{Al}}_{{0.12}} {\text{O}}_{{3}} \left( {{\text{Al-Brg}}} \right) + \left( {0.06x + 0.12} \right){\text{H}}_{{2}} {\text{O}}\\ &\quad \to 0.12{\text{ MgSi}}_{{{2}{-}x}} {\text{Al}}_{x} {\text{O}}_{{6}} {\text{H}}_{{{2} + x}} \left( {{\text{Al-phase D}}} \right) + \left( {0.94x- 0.12} \right){\text{MgO }} + \left( {1.06x- 0.24} \right){\text{SiO}}_{{2}}. \end{aligned}$$

Bolfan-Casanova et al.^30^ demonstrated that the major lower-mantle constituents, bridgmanite and ferropericlase (magnesiowüstite), can accommodate very little water, and completely denied the previous result^31^ that these nominally anhydrous phases could contain 0.2–0.4 mass% H_2_O component. Thus, the Al-phase D is a phase with potential as a reservoir of a large quantity of hydrogen in the normal lower-mantle.

MORB component of subducting slabs has Al_2_O_3_ content higher than pyrolite, and in the lower mantle bridgmanite with about 15–16 mass% Al_2_O_3_ component is stable in this composition^32,33^. This Al_2_O_3_ content is higher than that (about 8 mass%) in the bridgmanite composition employed in the present high-pressure experiments. Therefore, phase D in slabs would contain a large amount of Al_2_O_3_ component, and this content would be higher than that in the present Al-phase D. Indeed, super-aluminous phase D (simplified formula Al_2_SiO_6_H_2_), which could be stable over 2000 °C at 26 GPa (Ref.^9^), was produced from the similar Al-rich bulk composition^10^. If the incorporation of Al into phase D extends its stability field to higher pressure following the above crystal-chemical prediction, then Al-phase D in slabs can carry hydrogen much deeper in the lower mantle than previously estimated^7,34^. This speculation is consistent with the phase relation reported in the simplified system such as MgO–Al_2_O_3_–SiO_2_–H_2_O (Ref.^17^), but is inconsistent with that reported recently in the hydrous MORB system^35^. It was reported that in the former systems^17^ Al-phase D was stable up to ~ 55 GPa and Al-phase H was the stable hydrous phase at higher pressures, whereas in the latter system^35^ the stable region of Al-phase D was drastically reduced to ~ 25 GPa. This discrepancy in the stability relation of the hydrous phases may be attributed to the difference in the staring compositions including water contents, but the details remain to be solved. Even if Al-phase D decomposes into Al-phase H at lower pressures in actual subducting-slabs as suggested in the recent study^35^, hydrogen should be transported still deeper in the lower mantle by Al-phase H, stable up to much higher pressures^16^. The ultimately released water would be hardly absorbed into the surrounding lower-mantle constituents and be stored as hydroxyl groups in Al-phase D in the upper region of the lower mantle according to the reaction (1), although Al-phase H produced by reaction with bridgmanite might intervene depending on depth as will discussed below.

The role of Al-phase D in the dynamics of the lower mantle is especially noteworthy. The zero-pressure/room-temperature density of the present Al-phase D is calculated to be ρ_0_ = 3.35 g/cm^3^. Although the incorporation of Fe into Al-phase D can affect ρ_0_, the reported values (3.45–3.56 g/cm^3^) of Al-phase D containing some amount of FeO/Fe_2_O_3_ component (Mg_0.89−1.0_Fe_0.11−0.15_Al_0.03−0.32_Si_1.5−1.9_O_6_H_2.5−2.93_)^36–39^ are only a little higher than that of the present Al-phase D. These ρ_0_ values, including the present data, are all considerably lower than the representative value of the lower-mantle ρ_0_ = 4.15 g/cm^3^ (Ref.^40^). Therefore, a “wet-metasomatized” region containing Al-phase D would move upward owing to pronounced buoyancy even if it contains some amount of FeO/Fe_2_O_3_ component. Similarly, the ρ_0_ value of (Al, Fe)-free phase H is 3.38 g/cm^3^ (Ref.^41^), very close to that (3.43 g/cm^3^)^24^ of (Al, Fe)-free phase D; containing of some amount of Al_2_O_3_ and/or FeO/Fe_2_O_3_ components will not yield a significant increase in ρ_0_ of phase H as well [cf. ρ_0_ = 3.54 g/cm^3^ for δ-AlOOH (Refs.^42,43^); ρ_0_ = 4.45 g/cm^3^ for ε-FeOOH (Ref.^43^), isostructural with δ-AlOOH]. Thus, the same situation due to pronounced buoyancy would also occur in a “wet-metasomatized” region containing Al-phase H. This implies that Al-phase D and Al-phase H could be important agents for material transport in the lower mantle. These aluminous DHMS phases might have played an important role in extraction of water from the solid Earth to form the oceans.

Meanwhile, Al-phase D with much higher Al-content, such as super-aluminous phase D^9,10^, would be stable even in slabs subducting still deeper, but this would also decompose finally into Al-phase H, i.e. solid solutions between δ-AlOOH and MgSiO_4_H_2_ (phase H), with much higher Al-content to transport hydrogen presumably to the bottom of lower mantle^16^. In addition to such a super-aluminous phase H, recently discovered pyrite-type FeOOH_*x*_ (*x* ≤ 1)^44–46^, which could be produced in subducted banded-iron-formations, may also be a key to yielding the “wet-metasomatized” region under water-saturated conditions. This iron hydroxide is also promising as a hydrous phase stable at the bottom of lower mantle^44–46^ and probably forms solid solutions containing AlOOH and/or MgSiO_4_H_2_ components in deep subducted slabs^46^. A portion of water released by decomposition of the super-aluminous phase H and pyrite-type FeOOH_*x*_ due to heating at the core-mantle boundary may be spent on incorporating hydrogen into the outer core through the production of iron hydride FeH_*x*_. The remainder moving upward may contribute to yielding the water-saturated region in the lower mantle, and may migrate to the surface via Al-phase D from Al-phase H produced by reaction with bridgmanite. (In this process, Al-phase D is implicitly assumed to be formed in the upper region by reaction between Al-phase H and a high-pressure SiO_2_ polymorph.) Okuchi^47^ reported that hydrogen is highly-siderophile at high pressure conditions, and suggested that the core-materials (iron ponds) could incorporate a huge amount of hydrogen included in the magma ocean and the incorporated hydrogen never returns to the silicate Earth. If the Earth’s core has been saturated with hydrogen, it would have been released from the outer core over geologic time. However, the geophysical observations combined with the mineral physics data suggested that the core is undersaturated with hydrogen^48^. At present, hydrogen is thus unlikely to be provided from the outer core. In future, however, water continuously released from super-aluminous phase H and pyrite-type FeOOH_*x*_ might saturate the core with hydrogen. If so, hydrogen might come to be released from the outer core and this released hydrogen might also come to migrate to the surface via Al-phase D from Al-phase H.

## Methods

### High-pressure experiments

The high-pressure experiments were conducted using a 5000-ton Kawai-type multi-anvil apparatus^22^ installed at the Institute for Planetary Materials, Okayama University. The experimental procedures and techniques are essentially the same as those described in our previous studies^49–52^ as follows. We employed a 6 mm regular octahedron of sintered MgO containing 5% of Cr_2_O_3_ as a pressure-transmitting medium and a LaCrO_3_ as a heating material. The three runs reported here were performed under the different conditions shown in Table 1. The mixture of the starting materials, including an amount of liquid water suitable for over-saturation, was placed in a Pt capsule and sealed by arc-welding the capsule ends. In particular, liquid water was carefully injected into the capsule using a microsyringe. During arc-welding, the capsule was cooled by wrapping in water-soaked absorbent cotton to prevent evaporation of injected water. The Pt capsule was inserted into the LaCrO_3_ heater and electrically insulated from the heater by a MgO spacer. The heater was surrounded with ZrO_2_ thermal insulator, and then was put into the MgO octahedron. This cell assembly was set in the anvil assembly of tungsten carbide cubes with truncated edge lengths of 2 mm, and then was compressed up to the target pressure (26 or 27 GPa) at room temperature. The temperature was then raised to the target maximum temperature (1600 or 1900 °C) in each run at a rate of 35 °C/min. The temperature was controlled with a W97%Re3%–W75%Re25% thermocouple, whose junction was put at the midpoint of the outer surface of the Pt capsule. No correction was made for the pressure effect on emf. After being exposed to the different thermal history in each run (Table 1), the products were quenched at 1300 or 1600 °C by shutting off the electric power supply. The pressure was released slowly and the products were recovered at ambient conditions. The recovered samples were mounted with epoxy and polished for the chemical analyses using a JEOL JCMA-733II electron probe microanalyzer. For the analyses, the irradiated electron beam was focused to 5 μm in diameter, sufficiently smaller than area sizes of analyzed crystals, under operation conditions of a 15 kV acceleration voltage and a 10 nA beam current. No contamination from the cell assembly materials into the products was detected from qualitative electron probe microanalyses. For the phase identification, the polished samples were also characterized by a Rigaku RINT RAPID-R microfocus X-ray diffractometer with Cu Kα radiation (λ = 1.54184 Å) operated at 40 kV and 200 mA.

### Single-crystal X-ray diffraction intensity measurements and structure refinements

The single-crystal X-ray diffraction intensity measurements, data processing and structure refinements were conducted according to essentially the same procedures and techniques as those described in our previous studies^49–58^ as follows. A single crystal with a size of 75 × 45 × 20 μm^3^ produced in the run #3 was selected and then mounted on the tip of a glass fiber for X-ray diffraction intensity measurements using a graphite-monochromatized Mo *K*α radiation (λ = 0.71069 Å). The measurements were conducted at room temperature (296 K) using a Rigaku AFC-7R four-circle diffractometer operated at 60 kV and 250 mA. The unit-cell parameters were determined by the least-squares method from a set of 27 reflections within the range of 38° ≤ 2θ ≤ 50°. The intensity data of a total of 1961 reflections within 2° ≤ 2θ ≤ 100° were collected using the continuous ω–2θ scan mode and corrected for Lorentz-polarization factors and absorption effects (ψ-scan method). The unit-cell parameters were calculated as *a* = 4.8372(8) Å, *b* = 4.8359(11) Å, *c* = 4.3236(5) Å, α = 90.000(14)°, β = 90.005(12)° and γ = 119.981(12)° without any constraints, agreeing with a trigonal cell. The final unit-cell parameters were determined as *a* = 4.8379(4) Å and *c* = 4.3236(4) Å under the constraints of trigonal setting. Intensity statistics, indeed, showed Laue symmetry ${\bar{3}}$1*m* (trigonal cell). The intensity data were averaged in this Laue symmetry to give 351 unique reflections. Of these, unique reflections with $| {F_{{\text{o}}} }| \le 3{\upsigma }_{{\text{F}}}$ were eliminated, where ${\upsigma }_{{\text{F}}}$ is the standard deviation for observed structure factor $| {F_{{\text{o}}} } |$. Even if unique reflections had intensities of $| {F_{{\text{o}}} } | > 3{\upsigma }_{{\text{F}}}$ after averaging, those averaged from data set of equivalent reflections including reflection(s) with $| {F_{{\text{o}}} } | \le 3{\upsigma }_{{\text{F}}}$ were also discarded since these reflections were potentially affected by multiple scattering as in Refs.^51–56^. Finally, 167 unique reflections were used in the present refinements.

The crystal structure was determined by the direct method using the program SIR97 (Ref.^59^) and refined by minimizing the function $\sum {\upsigma }_{{\text{F}}}^{ - 2} ( {| {F_{{\text{o}}} } | - | {F_{{\text{c}}} } |} )^{2}$ using the full matrix least-squares program RADY^60^. Among the space groups subjected to Laue symmetry ${\bar{3}}$1*m*, the possible ones are *P*31*m*, *P*312 and *P*${\bar{3}}$1*m* because no systematic absences were observed. We selected the centrosymmetric space group *P*${\bar{3}}$1*m*, adopted in Al-free phase D^23–25^, because the structure refinements assuming the remaining two space groups resulted in unsuccessful convergence with larger reliability indices. Indeed, in the difference Fourier synthesis after the final refinement assuming *P*${\bar{3}}$1*m*, no significant residual electron densities were observed around the M, S and O sites; thus, site-splitting due to symmetry reduction to non-centrosymmetric subgroup *P*31*m* or *P*312 is most unlikely. H atom was excluded from the structure refinements because of its low X-ray scattering power. Scattering factors of Mg^2+^, Al^3+^, Si^4+^ (Table 6.1.1.3 in *International Tables for Crystallography*^61^), and O^2−^ (Tokonami^62^) were used. Anomalous dispersion coefficients for each scattering factor were taken from Table 4.2.6.8 in *International Tables for Crystallography*^61^. Several correction models for the secondary extinction effects were attempted during the refinements, and the isotropic correction of Type I^63,64^ with a Gaussian mosaic spread distribution model yielded the best fit.

In super-aluminous phase D^9,10^ (simplified formula Al_2_SiO_6_H_2_), the following three^10^ or four^9^ symmetrically distinct octahedral-sites are partially occupied by a disordered distribution of Al and Si: M-site (Wyckoff position 1*a*) and S-site (2*d*), which are also occupied in Al-free phase D^23–25^ (simplified formula MgSi_2_O_6_H_2_), and the one (2*c*)^10^ or two (2*c*, 1*b*)^9^ additional octahedral sites, which are vacant in Al-free phase D. The difference Fourier synthesis for the present Al-phase D, however, showed that no significant residual electron density peak is detected on these additional octahedral sites, which indicates that cations are distributed only on M- and S-sites as in Al-free phase D^23–25^. The structure refinements were therefore performed by varying *P*(^M^Mg) as the only valuable site occupancy parameter under the following constraints to keep the chemical composition from the electron probe microanalyses: *P*(^S^Si) ≡ 0.715 (fix), *P*(^S^Al) ≡ 0.135 (fix), *P*(^S^Mg) ≡ 0.625 − 0.5 × *P*(^M^Mg), where the superscripts M and S represent the occupied sites of the cations. The final structure refinement converged smoothly to *R* = 0.0320 and w*R* = 0.0319 with anisotropic displacement parameters. The resulting *P*(^M^Mg) is 0.979(8), indicating that M- and S-sites both are almost full occupied. In the final difference Fourier synthesis, the residual electron density peaks with a height of 0.36 eÅ^−3^ (Fig. 3d) were observed at equivalent positions of the coordinates (0.495, 0, 0.124), located 0.91 Å and 1.80 Å away from adjacent O atoms. These distances are reasonable as H–O (donor) and H···O (acceptor) bond lengths, respectively, which was also confirmed from the bond valence calculations^27^. We therefore assigned the peaks to H atoms.

The summary of crystallographic data, data-collection and refinement parameters is given in Supplementary Table S1. The refined structural parameters and the selected interatomic distances and angles are listed in Tables 3 and 4, respectively. Crystallographic Information File (CIF) is deposited in the Cambridge Structural Database (CSD) (Deposition No. 2118607).

## Supplementary Information

Supplementary Table S1.
