# Supplementary material for: Aluminous hydrous magnesium silicate as a lower-mantle hydrogen reservoir: a role as an agent for material transport
Source: Sci Rep. 2022 Mar 4;12:3594. doi: 10.1038/s41598-022-07007-8 (PMC8897469; doi:10.1038/s41598-022-07007-8)
Supplement: Supplementary file 1 — Supplementary Table S1. [file 41598_2022_7007_MOESM1_ESM.docx]

Supplementary Information

**Aluminous hydrous magnesium silicate as a lower-mantle hydrogen reservoir: a role as an agent for material transport**

Akihiko Nakatsuka^1*^, Akira Yoshiasa^2^, Makio Ohkawa^3^ & Eiji Ito^4^

^1^*Graduate School of Sciences and Technology for Innovation, Yamaguchi University, Ube 755-8611, Japan*

^2^*Faculty of Advanced Science and Technology, Kumamoto University, Kumamoto 860-8555, Japan*

^3^*Graduate School of Advanced Science and Engineering, Hiroshima University, Higashi-Hiroshima 739-8526, Japan*

^4^*Institute for Planetary Materials, Okayama University, Misasa 682-0193, Japan*

*Corresponding author. Email: tuka@yamaguchi-u.ac.jp

**This file includes:**

Supplementary Table S1

**Supplementary Table S1.**

Summary of crystallographic data, data-collection and refinement parameters.

| Chemical formula | Mg_1.25_Si_1.43_Al_0.27_O_6_H_2.97_ |
| --- | --- |
| Temperature (K) | 296 |
| Cell setting | Trigonal |
| Space group | *P*$\bar{3}$1*m* |
| *a* (Å) | 4.8379(4) |
| *c* (Å) | 4.3236(4) |
| *V* (Å^3^) | 87.64(2) |
| Crystal size (μm^3^) | 75 × 45 × 20 |
| Radiation used | Mo *K*α |
| Diffractometer | Rigaku AFC-7R |
| Monochromator | Graphite |
| Scan type | ω–2θ |
| 2θ_max_ (°) | 100 |
| Range of *h*, *k*, *l* | 0 ≤ *h* ≤ 10, −10 ≤ *k* ≤ 10, −9 ≤ *l* ≤ 9 |
| No. of measured reflections | 1961 |
| No. of unique reflections | 351 |
| No. of observed unique reflections  used in refinements [$\left\vert F_{o} \right\vert>3{}_{F}$] | 167 |
| No. of parameters | 13 |
| *R* | 0.0320 |
| w*R* | 0.0319 |
| Weighting scheme | ${}_{F}^{-2}$ |
